# Biomechanical study of posterior cruciate ligament tibial arrest avulsion fracture fixation with triple tibial channel net sutures

**DOI:** 10.1038/s41598-023-50479-5

**Published:** 2023-12-27

**Authors:** Yuan Li, Jun-Cai Liu, Ju Wu, Xu Peng, Guan-Jun Sun, Zhong Li, Yi Yin

**Affiliations:** 1 Department of Joint Surgery, Suining Central Hospital, Suining, 629000 Sichuan People’s Republic of China; 2 Department of Orthopaedics, Sichuan Provincial Laboratory of Orthopaedic Engineering, Affiliated Hospital of Southwest Medical University, Luzhou, 646000 Sichuan People’s Republic of China; 3 Department of Stomatology, Suining Central Hospital, Suining, 629000 Sichuan People’s Republic of China

**Keywords:** Diseases, Medical research

## Abstract

To investigate the biomechanical properties of posterior cruciate ligament avulsion fractures of the tibia fixed using four different methods, including triple tibial channel net suture fixation. In 40 porcine knees, a standardized bony avulsion of the posterior cruciate ligament was generated. Double tibial bone channel suture fixation was performed in group A, double-head hollow compression screw fixation was performed in group B, triple tibial bone channel net suture fixation was performed in group C, and cortical suspension EndoButton fixation was performed in group D. The constructs were cyclically loaded 500 times (10 to 100 N) to measure the initial displacement and stiffness values. Subsequently, loading to failure was performed, and the yield load and peak load were measured. The results were analysed by one-way ANOVA, with significance set at P < 0.05. The initial displacement in group D (1.00 ± 0.20 mm) was lower than that in group C (1.46 ± 0.33 mm, P = 0.000), group B (1.91 ± 1.71 mm, P = 0.000) and group A (3.91 ± 0.79 mm, P = 0.000), but there was no significant difference between groups B and C (P = 0.055). The initial stiffness in group A (50.59 ± 6.89 N/mm) was lower than that in group C (67.21 ± 12.80 N/mm, P = 0.001), group D (71.18 ± 9.20 N/mm, P = 0.000) and group B (78.67 ± 5.91 N/mm, P = 0.000). However, there was no significant difference between groups B and D or between groups C and D (P = 0.111 and P = 0.391). The yield load in group A (554.86 ± 71.43 N) was lower than that in group C (767.00 ± 34.53 N, P = 0.000), group D (777.62 ± 73.03 N, P = 0.000) and group B (837.50 ± 55.73 N, P = 0.000). There was no significant difference between groups C and D (P = 0.729). The peak load in group A (667.38 ± 61.54 N) was lower than that in group C (842.00 ± 26.20 N, P = 0.000), group D (867.63 ± 63.42 N, P = 0.000) and group B (901.25 ± 54.38 N, P = 0.000). There was no significant difference between groups C and D (P = 0.346). Different failure modes were found among the four groups. The triple tibial bone channel suture fixation group showed better initial stability and fixation strength, which was comparable to that in the cortical suspension EndoButton fixation group and double-head hollow compression screw fixation group and significantly stronger than that in the double tibial bone channel suture fixation group. This study analysed the dynamic and static indexes of posterior cruciate ligament tibial avulsion fractures fixed by four different fixation methods under cyclic loading tests and single failure loading tests, providing a theoretical basis for clinical treatment.

## Introduction

Posterior cruciate ligament (PCL) tibial avulsion fractures are a special type of fracture. The Meyers–McKeever classification system classifies PCL tibial arrest avulsion fractures into three types^1^. At present, there are many mature surgical methods for the treatment of Meyers–McKeever type II to type III PCL tibial avulsion fractures with arthroscopic reduction and internal fixation, but none of them can ensure that the physiological function, anatomical structure and biomechanical properties of the affected structures can be restored to the optimal state before surgery. Screws, washers and plate fixation are classic devices for fracture reduction and fixation^2^. Trickey et al.^3^ reported for the first time the treatment of PCL tibial avulsion fractures with open reduction and cancellous bone screw fixation. Screw fixation has shown good clinical results^4–6^; however, screw fixation requires a second operation to remove the implant and is not suitable for small fracture blocks. Arthroscopic suture fixation of tibial avulsion fractures can overcome the shortcomings of the above fixation methods and is currently a generally accepted treatment method^7,8^. The most commonly used of these minimally invasive fixation techniques is double tibial bone channel suture fixation. Some scholars have also adopted suture bridge fixation with wire anchors as a technique to achieve overall downwards compression fixation of fracture masses^9^, but this method is difficult to perform under arthroscopy, has a high surgical cost, and may stimulate the PCL after surgery. Second, the cortical suspension EndoButton fixation technology proposed in recent years can be used to achieve the elastic fixation of fracture blocks^10^, but the fracture blocks need to be sufficiently large. According to the advantages and disadvantages of the above fixation methods, this research group designed a method called triple tibial channel net suture fixation for treating type II–III PCL tibial interception avulsion fractures in the early stage and achieved good short-term clinical results. This method can be used to fix comminuted fractures under arthroscopy while causing almost no damage to the parenchymal part of the PCL and theoretically realizing planar fracture fixation. However, few biomechanical studies have been reported on the treatment of PCL tibial avulsion fractures with arthroscopic reduction and internal fixation^11^.

The purpose of this study was to investigate the biomechanical properties of PCL tibial avulsion fractures fixed using four different fixation methods, including triple tibial channel net suture fixation. It was hypothesized that triple tibial channel net suture fixation and cortical suspension EndoButton fixation could produce biomechanical properties comparable to those of double-head hollow compression screw fixation and that all three fixation methods are superior to double tibial bone channel suture fixation.

## Methods

### Specimens and preparation

**Figure 1:**
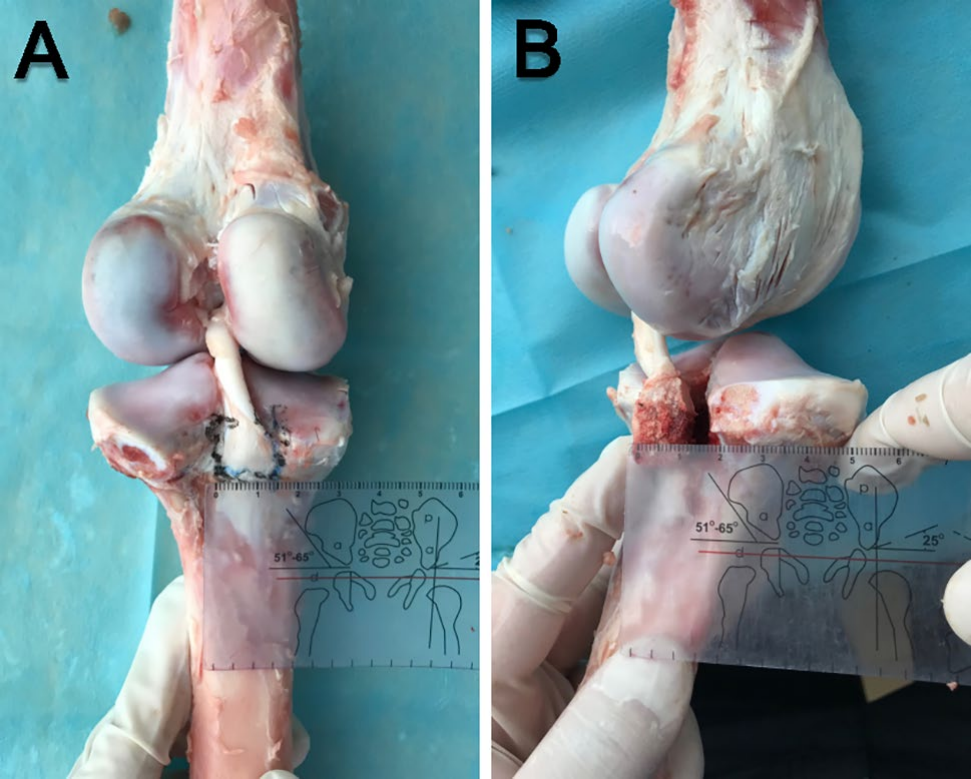
Preparation of the Meyers–McKeever type III fracture model.

The procedures performed in this study were approved by the ethics committee of Suining Central Hospital and were carried out in accordance with relevant guidelines and regulations. Additionally, the study was in compliance with the ARRIVE guidelines. A total of 32 fresh adult pigs ranging from 6 to 10 months old and weighing 120 to 150 kg were used. The femur and tibia were cut by hacksaw blades at a distance of 15 cm from the joint line. Bone mineral density (BMD) was measured on the tibial side of all samples by an ultrasonic bone densitometer to screen the knee joints for osteoporosis. First, all ligaments, meniscal tissue, excess soft tissue and structures other than the PCL were removed with a scalpel to fully expose the bone cortex. Second, the PCL footprint area was marked with a marker and then chiselled with a bone knife and bone hammer to make a thin rectangular tibial avulsion fracture model of a standardized size (18 mm (length) × 15 mm (width) × 8 mm (depth)). Finally, the size of the fracture block was measured and verified with a ruler. The modelling method is shown in Fig. 1.

### Study groups and fixation techniques

#### Group A

**Figure 2:**
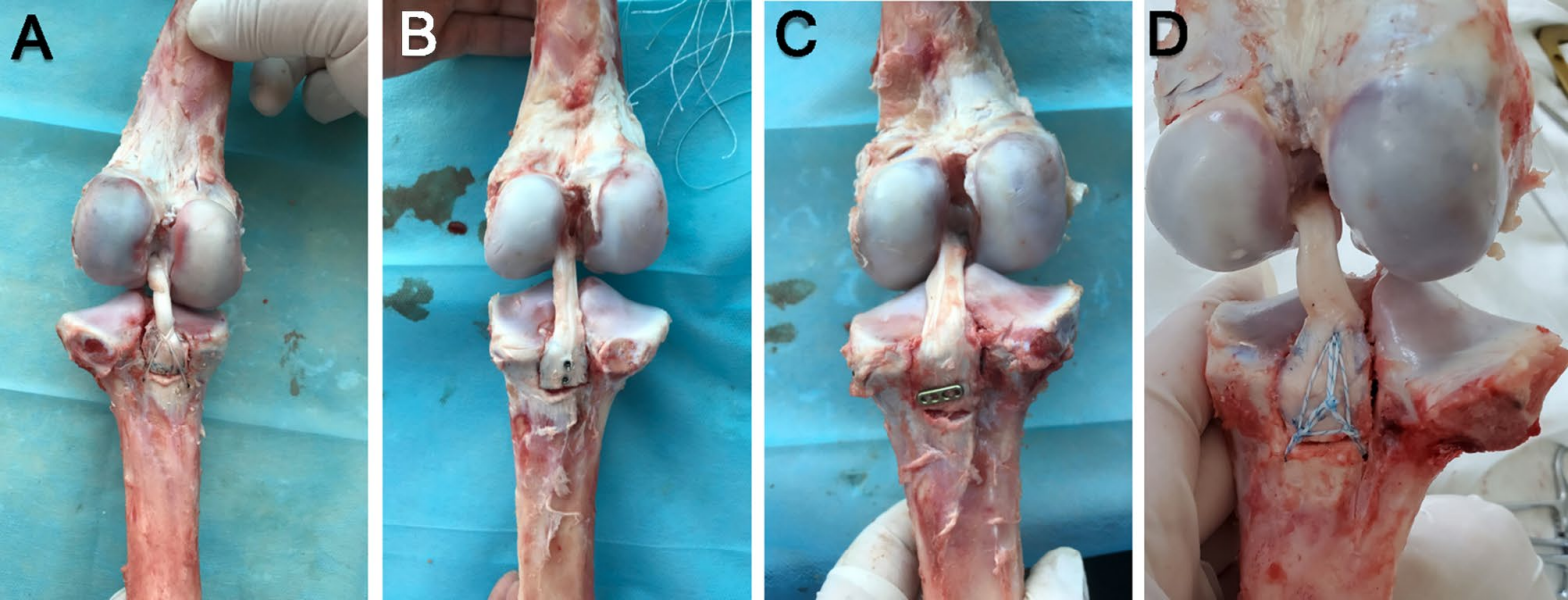
Fixation test groups: (**A**) double tibial bone channel suture fixation; (**B**) double-head hollow compression screw fixation; (**C**) triple tibial channel net suture fixation; (**D**) cortical suspension EndoButton fixation.

The fixation procedure is shown in Fig. 2. Two bone channels were made from the avulsion fracture surface of the tibial tubercle by an electric drill (with 2.0 mm Kirschner wire) and a PCL aiming device. The inner orifices of the two bone tunnels were located under and outside the tibial avulsion fracture surface, respectively, and the outer orifices of the two bone tunnels were located beside the tibial tubercles, with an interval of approximately 5 mm. Two 2.0 Ultrabraid sutures were used to cross the PCL near the tibial insertion point of the PCL in a “figure-of-eight”, and the four sutures were pulled out through the two bone tunnels and tightened at the exit for mutual knot fixation.

#### Group B

The fixation procedure is shown in Fig. 2. An electric drill (1.0 mm Kirschner wire) was used to drill into the tibia from the centre of the fracture surface almost perpendicular to the coronal plane of the tibia. The two holes were approximately 5 mm apart. Second, C-arm fluoroscopy of the pig knee joint was performed to determine the direction and position of the Kirschner wire. Finally, two 3.0 mm × 3.2 mm double-head hollow compression screws were driven into the avulsion fracture block by the guide pin with a screwdriver to complete fixation.

#### Group C

**Figure 3:**
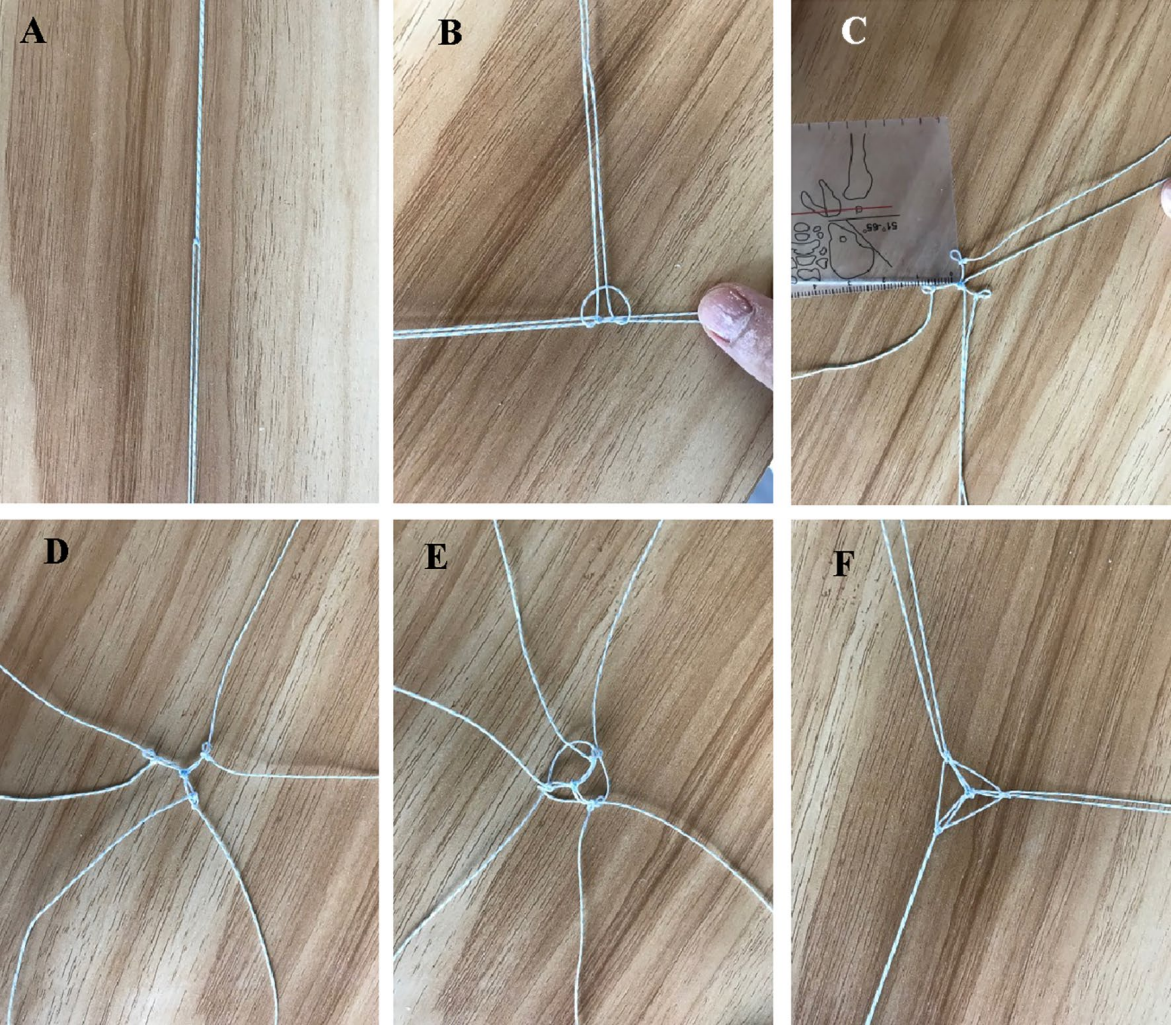
Net weaving: (**A**) Two high-strength sutures were folded in half, crossed, and tied in a knot. (**B,C**) A third suture is threaded into the previous knot, tied and fixed, resulting in the pattern shown in the figure after applying tension. (**D**) A slipknot was tied at any time in each root suture as the main line, with the slipknot approximately 0.5 cm away from the centre. (**E**) A secondary line was used to tie a slipknot on the main line and pulled tightly. (**F**) Then, each auxiliary line was threaded into the slipknot of the adjacent stitches and tightened again to form a triangular net.

The fixation procedure is shown in Fig. 2. Three tibial channels were made from the lateral fracture surface of the tibial tubercle by an electric drill (with 2.0 mm Kirschner wire) and a PCL aiming device. The inner orifices of the three tibial channels were located in three directions of the tibial fracture surface on the PCL footprint, and the outer orifices of the three bone canals were located beside the tibial tubercles, approximately 5 mm apart from each other. Then, the formed 2.0 Ultrabraid suture net (Fig. 3) was placed on the surface of the fracture block, the 6 tail lines were extracted from the three tibial canals, and two tail lines were extracted from each tunnel. The assistant pressed the fracture block to simulate the experimental direction of the front drawer to facilitate the restoration, and the surgeon tightened the external entrance of the tibial tunnels, knotted and sutured the lines together to complete the fixation.

#### Group D

The fixation procedure is shown in Fig. 2. First, the fracture block was manually reduced under direct vision, and two bone channels were made from the surface of the avulsion fracture block by an electric drill (with 2.0 mm Kirschner wire) and a PCL aiming device. Then, a 2.0 Ultrabraid suture was extracted through the tibial channel to the medial side of the tibial tuberculum through the lumbar puncture needle to attach an EndoButton titanium plate on the fracture surface after its position was adjusted. Finally, another EndoButton titanium plate was placed on the opposite side of the bone canal. The 2.0 Ultrabraid sutures were tightened and knotted to complete fixation.

### Tensile testing

**Figure 4:**
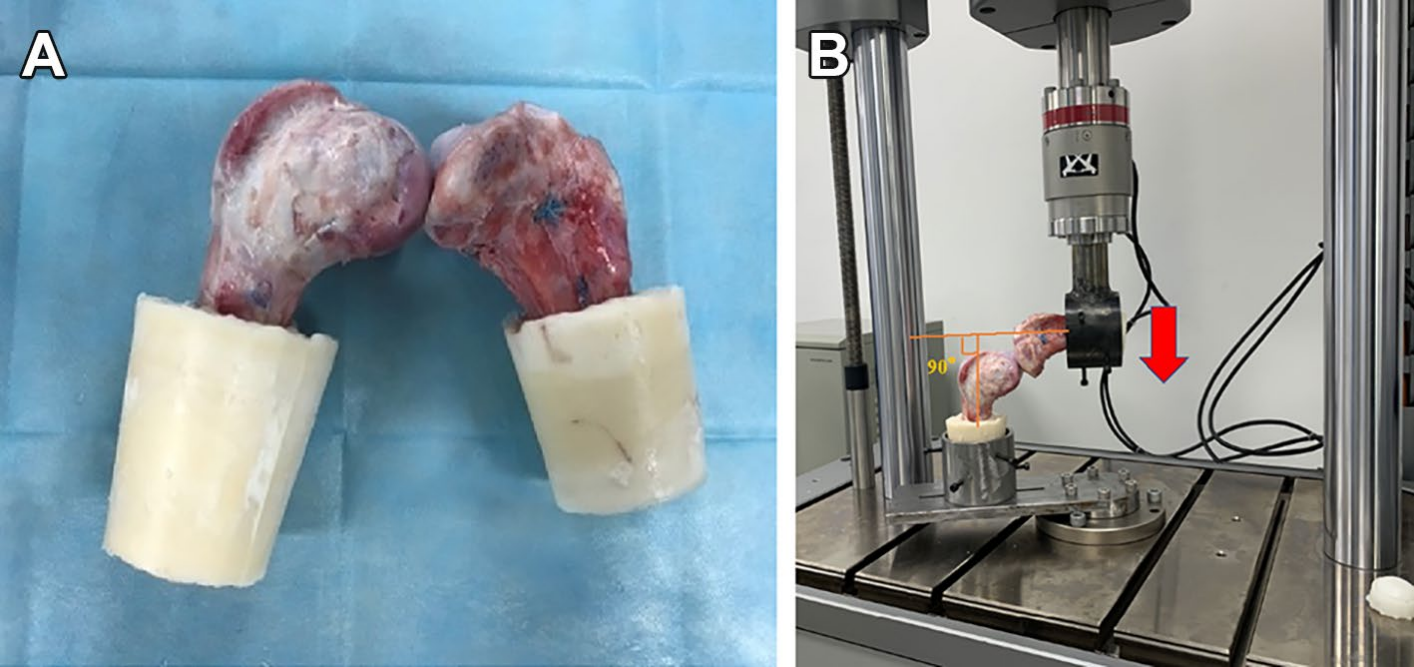
(**A**) Embedding of the femur and tibia in self-curing resin. (**B**) Fixed pig knee joint in the electronic dynamic and static universal testing machine at 90° of flexion (arrow indicates direction of force).

All samples were tested at room temperature, during which the samples were continuously moistened with normal saline. First, the tibial end and the femoral end were completely embedded in self-curing denture base resin powder and then firmly fixed with screws (Fig. 4). The tibial and femoral ends were then adjusted such that the pig knee specimens were flexed 90° in the biomechanical properties tester (Fig. 4). Sasaki^12^ and Wang^13^ reported that 90° of knee flexion was the best angle for PCL biomechanical tests. Before the test, the knee joint samples were pretreated with cyclic loading of 5–20 N 10 times, and the ligaments were tensioned. At this point, the displacement distance was set to 0 mm (Fig. 4).

### Cyclic loading test

After pretreatment, the samples were subjected to cyclic loading tests (1 Hz, 10–100 N, 500 cycles). This cyclic loading scheme was based on previous biomechanical testing schemes for avulsion fracture fixation techniques^10,14,15^. In the cyclic loading test, the primary index was the initial displacement, and the secondary index was the initial stiffness. The initial displacement describes the gradient of the strain associated with 10–100 N of force at the 10th and 500th repetitions of the cyclic loading process. The 10th repetition was chosen in this study because adjustment of the mechanical peak regulator is usually completed after the first 3 to 9 repetitions. The mean value of the 10th and 500th displacements was obtained by a computer to reflect the initial displacements of the fracture blocks in the process of fracture reduction, fixation and rehabilitation^16^. The slope of the load‒displacement curve in the 10th loading cycle was recorded to represent the initial stiffness value.

### Single failure loading test

After the cyclic loading test was completed, the tibia of each pig knee specimen was subjected to a single failure loading test at a speed of 200 mm/min until the internal fixation failed completely. During the experiment, the yield load and peak load were recorded. All internal fixation failures were recorded.

### Statistical analysis

All the data were analysed using SPSS 20.0 statistical software, and the quantitative data are presented as the mean ± standard deviation ($\overline{X }\pm S$). One-way ANOVA was used to compare data among groups, and the Kruskal‒Wallis H test was used to compare data with uneven variances and nonnormal distributions. The S–N–K method was used for pairwise comparisons between groups. The test level was α = 0.05, and *P* < 0.05 was considered to indicate a statistically significant difference. GraphPad Prism 6.0 was used for graphical analysis of the results.

## Results

### Basic data of the four groups

There was no significant difference in age, body weight or BMD among groups A, B, C and D (*P* > 0.05). There was also no significant difference in the length, width or depth of the avulsion fracture blocks among groups A, B, C and D (*P* > 0.05), as shown in Table 1.

**Table 1:** Basic data of the knee samples in the four groups.

Variable	A	B	C	D	Totality	*F*	*P*
Age, (m)	6.8 ± 1.0	7.0 ± 0.9	6.9 ± 1.1	7.0 ± 0.9	6.94 ± 0.9	0.042	0.988
Weight (kg)	138.6 ± 9.9	135.7 ± 10.0	131.6 ± 5.0	138.0 ± 8.4	135.33 ± 5.2	1.273	0.303
BMD (g/cm^3^)	1.18 ± 0.33	1.17 ± 0.14	1.17 ± 0.15	1.18 ± 0.67	1.18 ± 0.37	0.167	0.918
Length (cm)	2.0 ± 0.1	1.9 ± 0.2	2.0 ± 0.2	2.0 ± 0.2	2.0 ± 0.1	0.980	0.0392
Breadth (cm)	1.9 ± 0.1	1.8 ± 0.1	1.8 ± 0.1	1.9 ± 0.1	1.8 ± 0.1	0.636	0.539
Depth (cm)	0.9 ± 0.2	0.8 ± 0.2	0.9 ± 0.1	0.9 ± 0.1	0.9 ± 0.1	0.890	0.458

### Cyclic loading test

**Figure 5:**
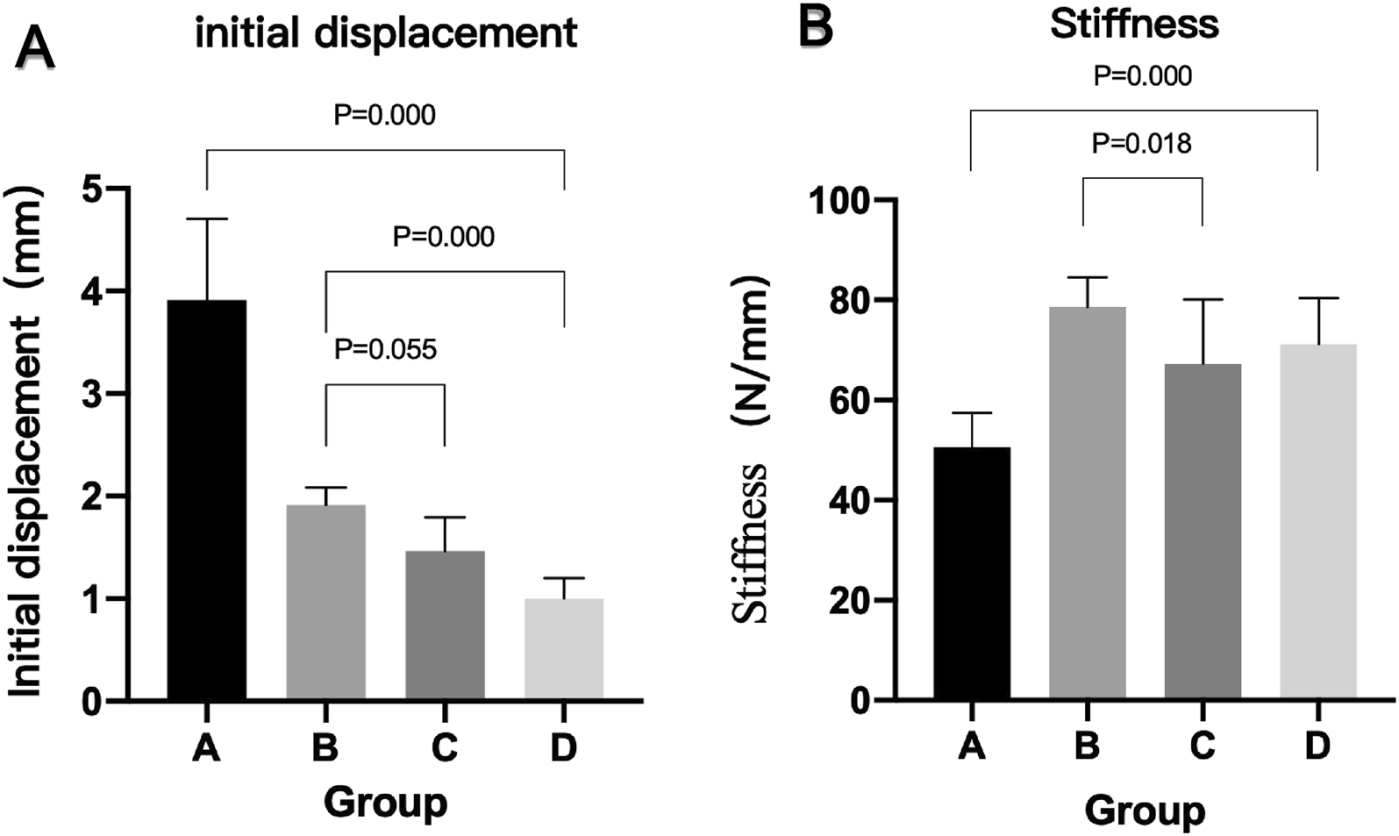
Histogram of the initial displacement and stiffness of the knee samples in the four groups.

All 32 pig knee samples underwent cyclic loading tests, during which the femur-PCL-tibia complex was intact and the avulsion fracture block remained stable in place. The findings are presented in Table 2 and Fig. 5. The initial displacement in group D was lower than that in group C (*P* = 0.000), group B (*P* = 0.000) and group A (*P* = 0.000), but there was no significant difference between groups B and C (*P* = 0.055). The initial stiffness in group A was lower than that in group C (*P* = 0.001), group D (*P* = 0.000) and group B (*P* = 0.000). However, there was no significant difference between groups B and D or between groups C and D (*P* = 0.111 and *P* = 0.391).

**Table 2:** Biomechanical test results.

Variable	A	B	C	D	*F*	*P*
Initial displacement (mm)	3.91 ± 0.79	1.91 ± 1.71	1.46 ± 0.33	1.00 ± 0.20	65.127	0.000
Stiffness (N/mm)	50.59 ± 6.89	78.67 ± 5.91	67.21 ± 12.80	71.18 ± 9.20	13.629	0.000
Peak load (N)	667.38 ± 61.54	901.25 ± 54.38	842.00 ± 26.20	867.63 ± 63.42	30.406	0.000
Yield load (N)	554.86 ± 71.43	837 ± 55.73	767.00 ± 34.53	777.62 ± 73.03	33.149	0.000

### Failure loading test results

**Figure 6:**
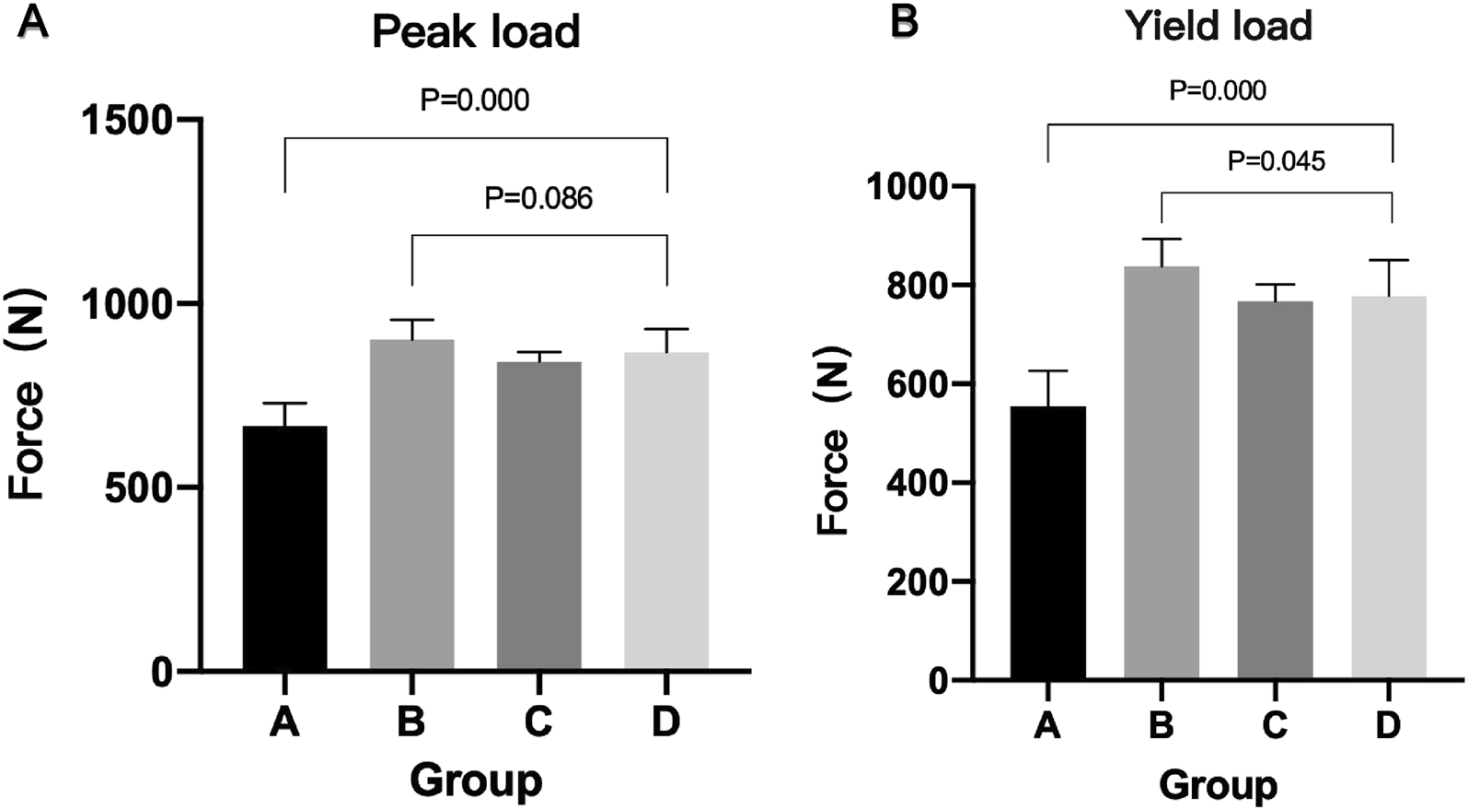
Histogram of the yield load and peak load of the knee samples in the four groups.

The findings are presented in Table 2 and Fig. 6. The yield load in group A was lower than that in group C (*P* = 0.000), group D (*P* = 0.000) and group B (*P* = 0.000). There was no significant difference between groups C and D (*P* = 0.729). The peak load in group A was lower than that in group C (*P* = 0.000), group D (*P* = 0.000) and group B (*P* = 0.000). There was no significant difference between groups C and D (*P* = 0.346). Different failure modes were found among the 4 groups.

### Sample failure types

**Figure 7:**
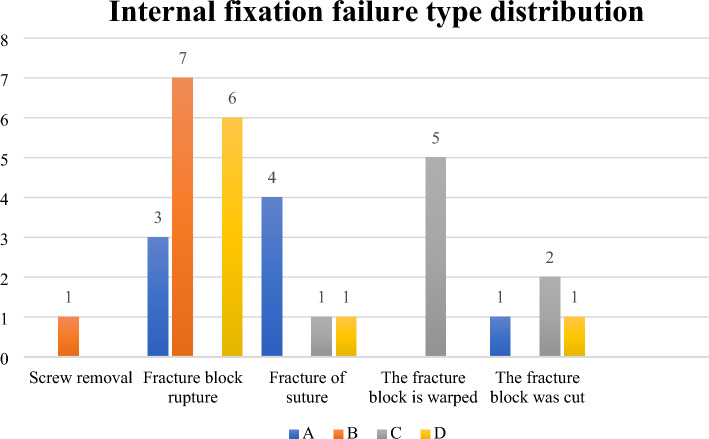
Distribution of failure types in four groups of experimental samples.

The types of internal fixation failure in the four groups of knee samples are shown in Fig. 7.

## Discussion

In biomechanical comparison of different fixation techniques, we found that the tibia three-bone suture fixation group showed better initial stability and fixation strength, which was comparable to that of double-channel suspension EndoButton and plate fixation and double-head compression hollow screw fixation and significantly stronger than double-channel suture fixation.

In this study, we compared the biomechanical properties of triple tibial channel net suture fixation with three other common fixation methods. The sizes of PCL tibial avulsion fracture models produced by different researchers are not uniform^17,18^. Considering clinical cases with the above reports, a standardized, thin rectangular model of a tibial avulsion fracture with an average fracture block size of 18 mm (L) × 15 mm (W) × 8 mm (D) was made in the PCL footprint area in this study. As there are currently no biomechanical studies evaluating the effect of different avulsion fracture volumes on fixation strength, future studies may provide a more detailed classification of PCL avulsion fractures based on factors such as fracture volume and avulsion degree, thereby allowing the development of more accurate and effective treatment plans. Screw fixation strength is correlated with BMD and trabecular structure^19,20^. Therefore, a bone density test was performed to confirm the equivalence of each group of specimens and reduce the influence of bone quality. The PCL in both pigs and humans is under the most tension at 90 degrees of knee flexion; thus, 90 degrees of knee flexion is used for biomechanical testing^21^. Although there are many recent reports in the literature about different techniques for the treatment of PCL tibial arrest avulsion fracture^10,11^, there are very few reports on the biomechanical properties of different fixation techniques. Sasaki et al.^12^ showed that arthroscopic suturing for PCL tibial avulsion was as reliable for initial fixation as open screw fixation. They only performed cyclic loading tests and did not perform single failure loading tests. In contrast, in the present study, a biomechanical comparison between cortical suspension EndoButton fixation and triple tibial channel net suture fixation was performed as well as single failure loading tests.

Open reduction and internal screw fixation is the most commonly used surgical fixation method for the treatment of PCL tibial avulsion fractures, with good early curative effects^2,11,22^. However, a second operation is needed to remove the internal fixation instrumentation, which can lead to a risk of impingement of the posterior femoral condyle. In addition, smaller fractures and comminuted fractures cannot be safely fixed by this method and are prone to further fracture. In this study, two double-headed hollow compression screws were used for fixation. Compared with hollow screws, nuts were easier to place and have a lower risk of intercondylar impact. Both Eggers et al.^23^ and Senekovic et al.^24^ showed that suture fixation provided greater fixation strength and initial stability than screw fixation. However, the results of this study showed that the initial displacement of the double-head compression hollow screw group was 1.91 ± 1.71 mm, which was significantly lower than that in the suture fixation group. The initial stiffness was 78.67 ± 5.91 N/mm, which was significantly greater than that in the suture group. The reasons for this difference is the sufficient volume of the fracture block fixed using two double-headed hollow compression screws and the greater resulting fixation strength, torsional and tensile resistance and stability. In addition, in the single failure loading test, the peak load in this group was 901.25 ± 54.38 N, which was similar to the fixed strength of groups C and D, and significantly higher than the 667.38 ± 61.54 N in the suture fixed group.

Orkel et al.^18^ compared the biomechanical properties of the modified suture bridge technique and the double-tunnel suture fixation technique and found that the initial stiffness at the beginning of the cyclic loading test (46.9 ± 3.9 N/mm vs. 40.8 ± 9.0 N/mm, P > 0.05) and the failure loading test was (286.8 ± 88.3 N vs. 234.3 ± 96.8 N, P > 0.05) was statistically similar. Although the former showed a higher initial stiffness during cyclic loading, in the context of postoperative rehabilitation, neither of the two fixation methods showed sufficient fixation strength to support rehabilitation training with high activity intensity in the early stage. However, Kosters et al.^25^ pointed out that the double tibial bone channel suture fixation method could still be considered. However, in clinical practice, double tibial bone channel suture fixation often leads to unstable reduction of type III rotating-displaced fractures, early postoperative relaxation, fracture block warping, and limited flexion. This study showed that the initial stability (1.46 ± 0.33 mm vs. 3.91 ± 0.79 mm, P = 0.000) and peak load (842.00 ± 26.20 N, vs. 667.38 ± 61.54 N, P = 0.000) of triple tibial channel net suture fixation were significantly higher than those of double tibial bone channel suture fixation. The experimental results indicate that triple tibial channel net suture fixation provides good initial stability and a strong biomechanical basis for early postoperative rehabilitation training. Except for the suture fixation group, there was no significant difference in the peak load or yield load among the other three fixation methods, indicating that a similar ability of these three fixation methods to treat an injury resulting from a single violent accident. In addition, the mesh suture used in this study has the following advantages: it can provide effective fixation for patients with small fractures and fragmentary fractures; it does not leave metal material in the joint cavity and thus does not require a second operation for removal. Compared with double tibial bone channel suture fixation, triple tibial channel net suture fixation has less of a damaging effect on the PCL itself and carries a lower risk of displacement.

Cortical suspension EndoButton fixation has shown outstanding clinical efficacy in the treatment of acromioclavicular joint dislocation and in cruciate ligament reconstruction and has been gradually introduced into PCL tibial avulsion fracture fixation^26–29^. In this study, we found that cortical suspension EndoButton fixation resulted in a higher failure load than double tibial bone channel suture fixation, which was no different from triple tibial channel net suture fixation; however, because of its ability to provide more stable initial fixation, it resulted in the lowest displacement under cyclic loading. Similar results were reported by Hapa et al.^10^. Cortical suspension EndoButton fixation offers better stability and allows higher intensity activity than double tibial bone channel suture fixation. Therefore, cortical suspension EndoButton fixation can allow patients to participate in more aggressive rehabilitation programs early after surgery. Similarly, Kosters et al.^25^ noted that EndoButton fixation with annular plate suspension exhibits biomechanical properties comparable to those of anterograde screw fixation and can be used as an alternative to conventional anterograde screw fixation. Clinically, the tension in cortical suspension EndoButton fixation can be dynamically adjusted according to the reduction of the fracture block, and a microplate of an appropriate size can be selected according to the size and type of the fracture block. In addition, the elasticity of the titanium plate allows for slight movement at the fracture site, in line with the biological principle of bone healing. However, it is not suitable for comminuted fractures.

As shown in this study, the biomechanical test data of the four different fixation methods fluctuated relatively widely, suggesting that early rehabilitation functional training still need to be individualized, even after mesh suture fixation. In addition, studies have reported the average strength of different ligaments and grafts^30^. However, there are no reports on the minimum strength needed for PCL reconstruction. Therefore, caution must be exercised when generalizing these biomechanical obtained using the porcine knee joint model to clinical treatment.

### Limitations

First, the number of samples is small, and there are some differences in bone density between pig knee joint specimens and adult knee joint specimens, thus, the biomechanical data are also different. Second, in this in vitro model, all fixation techniques were performed through an open approach under optimal conditions in which no other soft tissue was present, allowing for anatomical reduction and easy fixation of the fracture fragment. The accuracy of reduction and the resulting biomechanical properties may vary if the fracture fragment is fixed under arthroscopic or minimally invasive conditions. Third, this in vitro biomechanical study only reported initial stability and strength of fixation at a single angular position (90° of knee flexion), no experiments at other angles of knee flexion were performed, despite the use of a cyclic loading test protocol. Fourth, this study measured the failure load leading to complete stability of knee joint and PCL. Similar to previous studies, no complex rotational biomechanics tests or complex in vivo biomechanical tests were performed to test the PCL; thus, the results cannot reflect the stability of the PCL under different conditions, such as buckling, rotation, and other angles. Last, in this study, PCL tibial arrest avulsion fracture blocks were gouged en masse using special instruments rather than caused by trauma, so there may be differences in biomechanical properties.

## Conclusions

The triple tibial bone channel suture fixation group showed better initial stability and fixation strength, which was comparable to that in the cortical suspension EndoButton fixation group and double-head hollow compression screw fixation group and significantly stronger than that in double tibial bone channel suture fixation group.

## Data Availability

The datasets used and/or analysed during the current study are available from the corresponding author upon reasonable request.
